# Dephosphorylated NPr is involved in an envelope stress response of *Escherichia coli*

**DOI:** 10.1099/mic.0.000056

**Published:** 2015-05

**Authors:** Jaeseop Lee, Young-Ha Park, Yeon-Ran Kim, Yeong-Jae Seok, Chang-Ro Lee

**Affiliations:** ^1^​ Department of Biological Sciences, Myongji University, Yongin, Gyeonggido 449-728, Republic of Korea; ^2^​ Department of Biological Sciences and Institute of Microbiology, Seoul National University, Seoul 151-742, Republic of Korea; ^3^​ Department of Biophysics and Chemical Biology, Seoul National University, Seoul 151-742, Republic of Korea

## Abstract

Besides the canonical phosphoenolpyruvate-dependent phosphotransferase system (PTS) for carbohydrate transport, most *Proteobacteria* possess the so-called nitrogen PTS (PTS^Ntr^) that transfers a phosphate group from phosphoenolpyruvate (PEP) over enzyme I^Ntr^ (EI^Ntr^) and NPr to enzyme IIA^Ntr^ (EIIA^Ntr^). The PTS^Ntr^ lacks membrane-bound components and functions exclusively in a regulatory capacity. While EIIA^Ntr^ has been implicated in a variety of cellular processes such as potassium homeostasis, phosphate starvation, nitrogen metabolism, carbon metabolism, regulation of ABC transporters and poly-β-hydroxybutyrate accumulation in many *Proteobacteria*, the only identified role of NPr is the regulation of biosynthesis of the lipopolysaccharide (LPS) layer by direct interaction with LpxD in *Escherichia coli*. In this study, we provide another phenotype related to NPr. Several lines of evidence demonstrate that *E. coli* strains with increased levels of dephosphorylated NPr are sensitive to envelope stresses, such as osmotic, ethanol and SDS stresses, and these phenotypes are independent of LpxD. The C-terminal region of NPr plays an important role in sensitivity to envelope stresses. Thus, our data suggest that the dephospho-form of NPr affects adaptation to envelope stresses through a C-terminus-dependent mechanism.

## Introduction

The bacterial phosphoenolpyruvate : sugar phosphotransferase system (sugar PTS) is a group translocation system that mediates the translocation and concomitant phosphorylation of many sugars across the cytoplasmic membrane (Deutscher *et al.*, 2006). This system consists of two cytoplasmic general proteins, enzyme I (EI) and histidine phosphocarrier protein (HPr), which lack sugar specificity, and sugar-specific enzyme II (EII) components which usually have three domains, two cytosolic domains (EIIA and EIIB) and one membranous domain (EIIC) (Postma *et al.*, 1993). In addition to sugar uptake and phosphorylation, the sugar PTS plays important roles in the regulation of numerous metabolic processes by sensing the availability of nutrients. These regulatory functions include activation of adenylyl cyclase (Park *et al.*, 2006), inhibition of non-PTS sugar permeases (Deutscher *et al.*, 2006), chemoreception (Lux *et al.*, 1995), activation of the fermentation/respiration switch protein FrsA (Koo *et al.*, 2004; Lee *et al.*, 2011), activation of glycogen phosphorylase (Seok *et al.*, 1997), regulation of the σ^70^ activity (Park *et al.*, 2013) and inhibition of Mlc, the global repressor that controls the expression level of the sugar PTS and related proteins (Lee *et al.*, 2000; Nam *et al.*, 2001; Tanaka *et al.*, 2000).

Many Gram-negative bacteria have the so-called nitrogen PTS that parallels the sugar PTS. The nitrogen PTS constitutes another phosphoryl-transfer cascade whose relay proceeds sequentially from phosphoenolpyruvate (PEP) to EI^Ntr^ encoded by *ptsP*, NPr encoded by *ptsO* and EIIA^Ntr^ encoded by *ptsN*, which are homologous to the sugar PTS components EI, HPr and EIIA, respectively (Peterkofsky *et al.*, 2006; Pflüger-Grau & Görke, 2010; Powell *et al.*, 1995). Some Gram-negative bacteria such as *Pseudomonas putida* have all components of the nitrogen PTS, despite the lack of many sugar-specific EII components (Pflüger-Grau & Görke, 2010; Pflüger and de Lorenzo, 2008). The *ptsO* and *ptsN* genes are located in the same operon with *rpoN* encoding σ^54^ controlling nitrogen-related genes, and this operon also contains the genes encoding LptB, a component of an ABC transporter for lipopolysaccharide (LPS) (Sperandeo *et al.*, 2007) and RapZ (an RNase adaptor protein for degradation of GlmZ, a small RNA regulating cell wall biosynthesis) (Göpel *et al.*, 2013; Kalamorz *et al.*, 2007). Expression of the *rpoN* operon is under the control of σ^E^ as well as σ^70^ (Rhodius *et al.*, 2006). Since no phosphate acceptor of EIIA^Ntr^ has yet been demonstrated, the nitrogen PTS appears to function mainly in regulation. EIIA^Ntr^ regulates a variety of processes including potassium homeostasis in *Escherichia coli* and *Rhizobium leguminosarum* (Lüttmann *et al.*, 2009; Lee *et al.*, 2007; Prell *et al.*, 2012), sigma factor selectivity in *E. coli* (Lee *et al.*, 2010), nitrogen metabolism in some species including *Klebsiella pneumonia* (Merrick & Coppard, 1989; Powell *et al.*, 1995), phosphate starvation in *E. coli* (Lüttmann *et al.*, 2012), regulation of many ATP-dependent ABC transporters in *Rhizobium leguminosarum* and *Bradyrhizobium japonicum* (King & O’Brian, 2001; Prell *et al.*, 2012), virulence of several pathogenic bacteria such as *Legionella pneumophila*, *Salmonella enterica* and *Brucella melitensis* (Choi *et al.*, 2010; Dozot *et al.*, 2010; Higa & Edelstein, 2001), poly-β-hydroxybutyrate accumulation in *Azotobacter vinelandii*, *P. putida* and *Ralstonia eutropha* (Kaddor & Steinbüchel 2011; Segura & Espín, 1998; Velázquez *et al.*, 2007), carbon metabolism in *E. coli* and *Pseudomonas* species (Chavarría *et al.*, 2012; Powell *et al.*, 1995) and regulation of ppGpp accumulation in *Ralstonia eutropha* (Karstens *et al.*, 2014). These pleiotropic effects of EIIA^Ntr^ imply the physiological importance of the nitrogen PTS. Notably, although two components of the nitrogen PTS, EIIA^Ntr^ and NPr, are located in the same operon with *rpoN*, the role of this system related to nitrogen metabolism has been challenged (Ninfa, 2011; Reaves & Rabinowitz, 2011). However, two recent studies showed that the phosphorylation state of the nitrogen PTS is regulated by the availability of a preferred nitrogen source in *E. coli* and *Sinorhizobium meliloti* (Goodwin & Gage, 2014; Lee *et al.*, 2013). Therefore, elucidation of the molecular basis for regulatory roles of the nitrogen PTS in nitrogen metabolism is required.

Despite many reports about the function of EIIA^Ntr^ in various bacteria, the only identified role of NPr is the regulation of lipid A biosynthesis (Kim *et al.*, 2011). The dephosphorylated form of NPr decreased lipid A biosynthesis through a direct interaction with LpxD, which is an enzyme catalysing the second acylation of UDP-glucosamine, the third step in lipid A biosynthesis (Bartling & Raetz, 2008). Therefore, we assumed that NPr may play multiple physiological roles in *E. coli*.

In this report, we demonstrate the connection between dephosphorylated NPr and an envelope stress response. Cells with increased levels of dephosphorylated NPr were sensitive to osmotic, ethanol and SDS stresses, and these phenotypes were independent of NPr-mediated LpxD inhibition. The C-terminal region of NPr is an important determinant for sensitivity to these stresses. Thus, we propose that the dephosphorylated form of NPr negatively regulates the adaptation of cells to envelope stress through an unknown but C-terminus-dependent mechanism.

## Methods

### 

#### Bacterial strains, plasmids, and culture conditions.

The bacterial strains and plasmids used in this study are listed in Table S1, available in the online Supplementary Material. Bacterial cells were grown as described previously (Lee *et al.*, 2010). The *ptsN* deletion mutant was constructed using *E. coli* DY330 as described previously (Yu *et al.*, 2000). The *ptsN* gene (from the start codon to the stop codon) was replaced by the ampicillin-resistance gene (Amp^R^). The ampicillin-resistance gene was amplified by PCR from the pRE1 plasmid with the following primers: forward primer, 5′-TGCTCCGAGCCTGTTCCACTGTTTGAGTGGGCAGGTTCTTAGGTGAAATTATGAGTATTCAACATTTCCG-3′ and reverse primer, 5′-ACCATGTACTGTTTCTCCTCACAACGTCTAAAAGAGACATTACCGAATAATTACCAATGCTTAATCAGTG-3′. The double mutants of the nitrogen PTS genes were constructed by P1 transduction of the antibiotic-resistant gene region. All plasmids were constructed using standard PCR-based cloning procedures and verified by sequencing. To construct pCR2HN, in which expression of NPr tagged with six histidines at its N terminus (His-NPr) is under the control of the pRE1-vector system, the pNPr plasmid was digested with *Nde*I and *Bam*HI, and the fragment encoding *ptsO* was cloned into pRE1-His-Tag (Zhu *et al.*, 1997). To construct pCR2HC, the vector for expression of NPr tagged with six histidines at its C terminus (NPr-His), a forward primer possessing a synthetic *Nde*I site (underlined) in the ATG start codon (in boldface type) of the *ptsO* gene (5′-AACGTAACAT **ATG**ACCGTCAAGCAAACTGT-3′) and a reverse primer with a synthetic *Bam*HI site (underlined) downstream of six histidine codons (5′-AAAGTGAGGATCC **TTA**GTGGTGGTGGTGGTGGTGATCTTCATCAAAACC-3′) (the stop codon in bold type) were used to amplify the *ptsO* gene from MG1655 genomic DNA. After digestion, the *Nde*I–*Bam*HI fragment was inserted into the corresponding sites of pRE1 (Reddy *et al.*, 1989). The expression vector pNPr(H16A), for overproduction of NPr(H16A), was generated using an additional mutagenic primer pair covering the region coding for His16: forward primer, 5′-AACAAGCTGGGCATGGCTGCCCGGCCTGCA-3′; reverse primer, 5′-TGCAGGCCGGGCAGCCATGCCCAGCTTGTT-3′ (changed bases underlined). To construct pCR2HN85(H16A), which expresses His-NPr(H16A) truncated of the five C-terminal residues, the forward primer of the *ptsO* gene and a reverse primer with the synthetic *Bam*HI site (underlined) (5′-ATTAATCGGATCC **TTA**AGAATTAAAGAGGG-3′) (a new stop codon in bold type) were used to amplify the truncated *ptsO* gene from the pNPr(H16A) plasmid. After digestion, the *Nde*I–*Bam*HI fragment was inserted into the corresponding sites of pRE1-His-Tag (Zhu *et al.*, 1997). Similarly, the pCR2HN(H16A,ELE) plasmid was constructed using the forward primer of the *ptsO* gene and a reverse primer (5′-TGAGGATCC **TTA**CTCGAGTTCAGAATTAAA-3′), which has the three codons (CTCGAGTTC) encoding three residues (ELE) at the C-terminal end of HPr to replace the five C-terminal residues with the three residues of HPr. To construct pEI^Ntr^(H356A), a mutagenic primer pair covering the region coding for His356, a forward primer (5′-GGCGCAGCCAACTCCGCTGCTGCGATCATG-3′) and a reverse primer (5′-CATGATCGCAGCAGCGGAGTTGGCTGCGCC-3′) (changed bases underlined), was used. Similarly, plasmids for overexpression of point mutant proteins of five residues (GFDED) in the C terminus of NPr were generated using mutagenic primer pairs: D90A-F (5′-TCTGGTTTTGATGAAGCTTAATCTTCATCA-3′), D90A-R (5′-TGATGAAGATTAAGCTTCATCAAAACCAGA-3′), E89A-F (5′-TCTGGTTTTGATGCAGACTAGTCTTCATCA-3′), E89A-R (5′-TGATGAAGACTAGTCTGCATCAAAACCAGA-3′), D88A-F (5′-AATTCTGGTTTTGCTGAGGATTAATCTTCA-3′), D88A-R (5′-TGAAGATTAATCCTCAGCAAAACCAGAATT-3′), F87A-F (5′-CTCTTTAATTCTGGCGCCGATGAAGATTAA-3′), F87A-R (5′-TTAATCTTCATCGGCGCCAGAATTAAAGAG-3′), G86A-F (5′-GCCCTCTTTAATTCTGCTTTTGATGAAGAT-3′) and G86A-R (5′-ATCTTCATCAAAAGCAGAATTAAAGAGGGC-3′) (changed bases underlined). The expression vector pRE1-LpxD, for overproduction of LpxD, was generated using a primer pair: LpxD-F, 5′-TAAATAACATATGCCTTCAATTCGACTGGC-3′ (*Nde*I site underlined) and LpxD-R, 5′-GAACAAAGGATCCAACGTTAGTCTTGTTGA-3′ (*Bam*HI site underlined). To remove the internal *Nde*I site within the *lpxD* gene, we designed an additional primer pair covering the internal *Nde*I site: LpxD-LK-F, 5′-CGTAATCAACGGGCTTATGGAAATATGCGA-3′ and LpxD-LK-R, 5′-AGCCCGTTGATTACGCTGGCTCCGCCGATC-3′ (changed bases underlined). We carried out the first PCRs to amplify the *lpxD* gene from MG1655 genomic DNA using the LpxD-F/LpxD-LK-R pair and the LpxD-R/LpxD-LK-F pair. The mixture of first PCR products was used as template for the second PCR using the LpxD-F/LpxD-R pair. The second PCR product was digested with *Nde*I and *Bam*HI and the fragment encoding *lpxD* was cloned into pRE1.

#### Reverse transcription (RT)-PCR.

The transcript levels of *lpxD* were analysed by RT-PCR with primers specific for *lpxD* or 16S rRNA. The total RNA was extracted using the RNeasy mini kit (Qiagen) according to the manufacturer’s instructions from cells grown to mid-exponential phase in LB medium with or without 1 mM IPTG. The preparations were treated with RNase-free DNase (Promega) at 37 °C for at least 1 h to eliminate contaminating DNA. The absence of contaminating genomic DNA in RNA preparations was verified by PCR. The same amount of RNA from each culture was converted into cDNA using the cDNA EcoDry Premix (Clontech). The cDNAs were diluted 10-fold and subjected to RT-PCR analyses using *lpxD-*specific primers: forward primer, 5′-ATGCCTTCAATTCGACTGGCTGATTTAGCG-3′; reverse primer, 5′-GCAACCGGCACCGATAATCACGTTATCGCC-3′. The 16S rRNA transcript was used as a loading control. The amplification reactions were performed in a GeneAmp PCR System for 5 min at 94 °C, followed by 15 (16S rRNA) or 25 cycles (*lpxD*) of 94 °C for 20 s, 55 °C for 20 s and 72 °C for 1 min per kb, concluding with extension at 72 °C for 4 min. The transcript levels of *ptsO* were analysed by a similar manner using a *ptsO*-specific primer pair: forward primer, 5′-ATGACCGTCAAGCAAACTGTTGAAATCACA-3′; reverse primer, 5′-AAACCAGAATTAAAGAGGGCGATAACGGCG-3′.

#### Western blotting.

To determine the intracellular levels of mutant NPrs, we made polyclonal antibodies against NPr using female ICR mice. Cells were grown in LB medium to mid-exponential phase and 0.4 ml of cell culture was collected. After boiling for 5 min, the samples were analysed with 15 % SDS-polyacrylamide gels. Immunoblotting was performed according to standard procedures using specific antibodies.

#### Isolation of LPS.

LPS was isolated as described previously (Kim *et al.*, 2011). Briefly, cells were grown to stationary phase in 10 ml LB medium at 30 °C and harvested by centrifugation. After washing, the cells were collected in a 1.5 ml tube by centrifugation. LPS was recovered in an insoluble form by boiling in a solution containing 10 mM Tris/HCl (pH 8), 50 mM MgCl_2_ and 2 % Triton X-100 for 15 min. After cooling at room temperature for 10 min, the mixture was centrifuged at 16 000 ***g*** at 25 °C for 15 min. LPS in the pellet was solubilized by incubating with shaking in a solution containing 50 mM EDTA and 2 % Triton X-100 at 37 °C for 4 h. The suspension was then centrifuged at 16 000 ***g*** at 37 °C for 15 min and the supernatant solution was transferred to a fresh tube. To reprecipitate LPS, the supernatant was mixed with MgCl_2_ (final concentration 150 mM), incubated at 37 °C for 3 h and centrifuged at 20 000 ***g*** for 90 min at 37 °C. The transparent LPS precipitate was resuspended in 2× SDS sample buffer and boiled for 5 min. Aliquots of the samples were analysed by SDS-PAGE and visualized by silver staining.

## Results

### Effect of the nitrogen PTS on salt stress

**Fig. 1.**
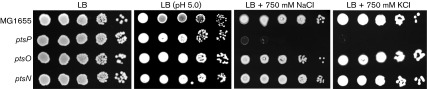
Effect of the nitrogen PTS on salt stress. Stationary-phase cells of the indicated strains grown in LB medium were serially diluted 10-fold from 10^8^ to 10^4^ cells ml^−1^ and 2 µl aliquots were spotted onto a LB plate (LB), a LB plate buffered at the pH 5.0 using 100 mM sodium citrate (LB pH 5.0), a LB plate supplemented with 750 mM NaCl (LB+750 mM NaCl) or a LB plate supplemented with 750 mM KCl (LB+750 mM KCl). After incubation at 37 °C for 14–20 h, the plates were scanned.

One of physiological roles of the nitrogen PTS is the maintenance of potassium homeostasis through regulating TrkA and KdpD (Lüttmann *et al.*, 2009; Lee *et al.*, 2007). Potassium is implicated in diverse processes such as homeostasis of cytoplasmic pH, the adaptation to osmotic conditions, the activation of cytoplasmic enzymes and the maintenance of cell turgor (Epstein, 2003). To know the relationship between the nitrogen PTS and these potassium-mediated cellular effects, we tested effects of the nitrogen PTS on pH and salt stresses. Mutant strains deleted for the nitrogen PTS genes did not show any significant difference in growth compared to WT when grown in LB medium or under acid stress conditions (Fig. 1). However, deletion of EI^Ntr^ (encoded by *ptsP*), the first enzyme in the phosphoryl-transfer cascade of the nitrogen PTS, significantly decreased salt tolerance of MG1655 cells in the LB medium containing 750 mM NaCl, whereas the deletion of the *ptsO* or *ptsN* gene hardly affected the sensitivity to salt stress (Fig. 1). Hypersensitivity to salt stress in the *ptsP* mutant was also exhibited when KCl was used instead of NaCl, implying that this phenotype was independent of potassium homeostasis. Recently, a growth defect of the *ptsN* mutant on certain organic nitrogen sources was shown to be observed only in *E. coli* strains lacking a functional *ilvG* gene (Reaves & Rabinowitz, 2011). The *ilvG* gene encodes a valine-insensitive acetohydroxy acid synthase (AHAS) II which catalyses the first common step in the biosynthetic pathway of the three branched-chain amino acids. To clarify whether a growth defect of the *ptsP* mutant on salt stress was dependent on the *ilvG* genotype, we constructed a *ptsP* mutant strain in the *ilvG* ^+^ genetic background and tested the effect on salt stress. As shown in Fig. S1, the *ptsP* mutant having a functional *ilvG* gene was also hypersensitive to salt stress, suggesting that this phenotype of the *ptsP* mutant was independent of the *ilvG* genotype.

### The dephosphorylated form of NPr increases sensitivity to salt stress

**Fig. 2.**
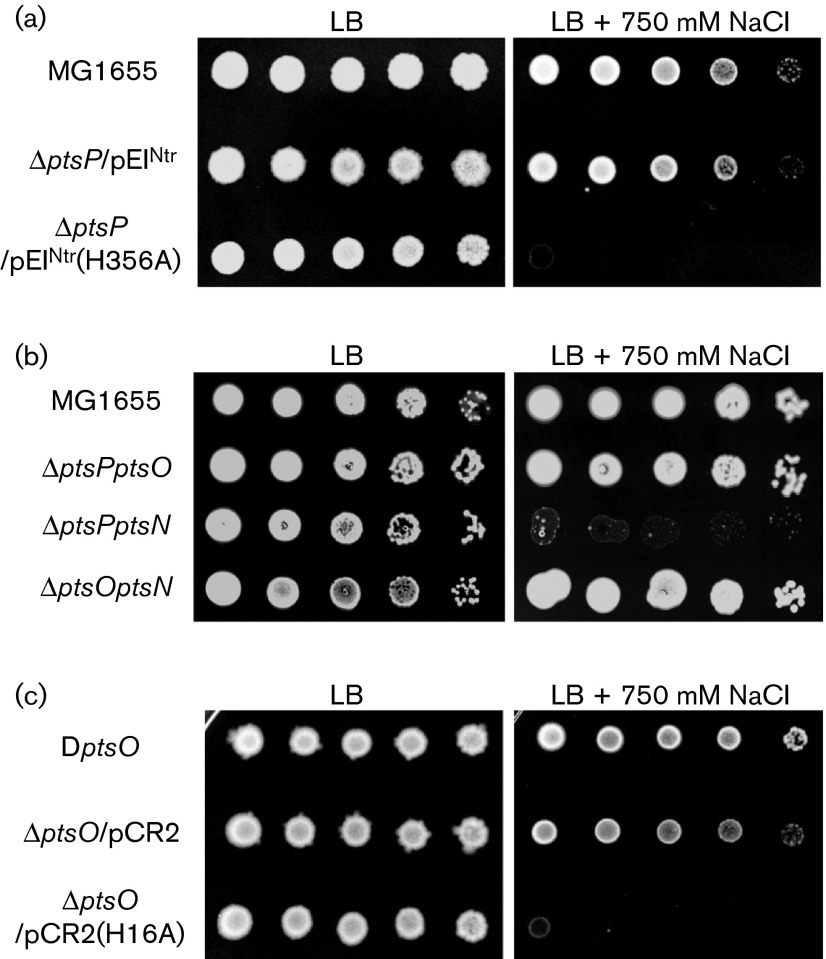
Salt hypersensitivity of cells with different levels of dephosphorylated NPr. Stationary-phase cells of the indicated strains were serially 10-fold diluted from 10^8^ to 10^4^ cells ml^−1^ and spotted onto LB plates with and without the addition of 750 mM NaCl as indicated.

To elucidate a cellular mechanism for the salt-sensitive phenotype of the *ptsP* mutant, we first checked whether the presence of the phospho-form of EI^Ntr^ was necessary for the growth of *E. coli* cells in salt stress. We constructed a pRE1-based plasmid, pCR1(H356A), expressing a mutant form of EI^Ntr^ in which the phosphorylatable His356 residue was mutated to Ala. Although the *ptsP* mutant transformed with pCR1 expressing wild-type EI^Ntr^ could grow in the LB medium containing 750 mM NaCl to a level of WT cells, the growth defect of the *ptsP* mutant under salt stress could not be recovered by expression of EI^Ntr^(H356A) (Fig. 2a). This indicated that the phospho-form of EI^Ntr^, but not the dephospho-form, was necessary for resistance of cells to salt stress.

The sequential phosphoryl-transfer cascade of the nitrogen PTS is as follows: PEP→EI^Ntr^→NPr→EIIA^Ntr^ (Rabus *et al.*, 1999). To determine whether salt hypersensitivity of the *ptsP* mutant was related to the phosphotransfer ability of EI^Ntr^ or phosphorylated EI^Ntr^ itself, we analysed the growth rates of mutants lacking two of the three nitrogen PTS genes. Notably, although the *ptsP ptsN* double mutant was extremely sensitive to salt stress like the *ptsP* mutant, cells of the *ptsP ptsO* double mutant strain and the *ptsO ptsN* double mutant strain exhibited normal growth rates, comparable to that of the WT strain in LB medium supplemented with 750 mM NaCl (Fig. 2b). Therefore, these results implicate that the phosphotransfer ability of EI^Ntr^ was necessary for salt stress resistance of *E. coli* cells and the dephospho-form of NPr negatively affected the adaptation to salt stress.

To confirm this idea, we constructed the pCR2(H16A) plasmid, expressing a mutant form of NPr which cannot be phosphorylated by EI^Ntr^. When the *ptsO* mutant was transformed with pCR2 (Lee *et al.*, 2005) expressing WT NPr, plasmid-harbouring cells also exhibited a normal growth rate like the *ptsO* mutant. However, cells expressing NPr(H16A) were as sensitive to salt stress as the *ptsP* mutant (Fig. 2c). The same result was obtained when NPr(H16A) was expressed in the WT strain (Fig. S2), suggesting that dephosphorylated NPr negatively affected the adaptation to salt stress and the salt-sensitive phenotype of the *ptsP* mutant was due to an increased level of dephospho-NPr.

### Hypersensitive phenotype of the *ptsP* mutant to salt stress is independent of NPr-mediated LpxD inhibition

**Fig. 3.**
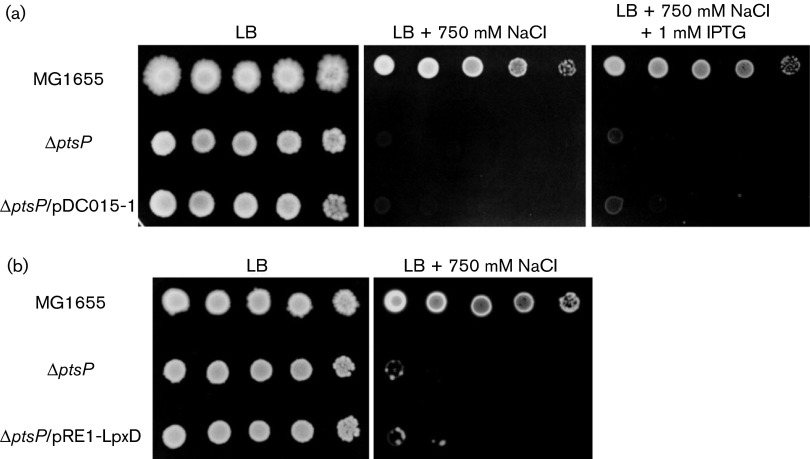
Effect of LpxD overexpression on salt hypersensitivity of the *ptsP* mutant. (a) Stationary-phase cells of the indicated strains grown in LB medium were serially diluted 10-fold from 10^8^ to 10^4^ cells ml^−1^, and 2 µl aliquots were spotted onto a LB plate, a LB plate supplemented with 750 mM NaCl, or a LB plate supplemented with 750 mM NaCl and 1 mM IPTG as indicated. (b) Stationary-phase cells of the indicated strains grown in LB medium were serially diluted 10-fold from 10^8^ to 10^4^ cells ml^−1^, and 2 µl aliquots were spotted onto LB plates with and without the addition of 750 mM NaCl as indicated.

In a previous report, it was shown that dephosphorylated NPr inhibits LpxD by direct interaction (Kim *et al.*, 2011). LpxD catalyses the third step of lipid A biosynthesis, the acylation of UDP-3-*O*-(*R*-3-hydroxymyristoyl)-glucosamine. Because an increase of dephosphorylated NPr can inhibit the LpxD activity, we assumed that sensitivity of the *ptsP* mutant to salt stress might be due to a decreased LpxD activity. To verify this assumption, we examined whether overexpression of LpxD could restore the growth of the *ptsP* mutant under salt stress conditions. The expression level of *lpxD* was significantly increased in the *ptsP* mutant cells harbouring the pET11d-based LpxD expression vector pDC015-1 (Bartling & Raetz, 2008), compared with that of WT or the *ptsP* mutant, and it could be further induced by the addition of 1 mM IPTG (Fig. S3a). However, salt sensitivity of these cells was hardly affected by the LpxD level (Fig. 3a). To reconfirm these results, we constructed a pRE1-based LpxD expression plasmid, pRE1-LpxD. The *ptsP* mutant transformed with pRE1-LpxD also exhibited the phenotype as sensitive to salt stress as the *ptsP* mutant (Fig. 3b), despite overexpression of the *lpxD* gene and the sufficient synthesis of LPS (Fig. S3b, c). Therefore, these data indicate that the sensitivity to salt stress caused by an increased level of dephosphorylated NPr was independent of NPr-mediated LpxD inhibition.

### The salt hypersensitivity of strains with increased levels of dephosphorylated NPr is not restored by overexpression of the *osmY* gene

**Fig. 4.**
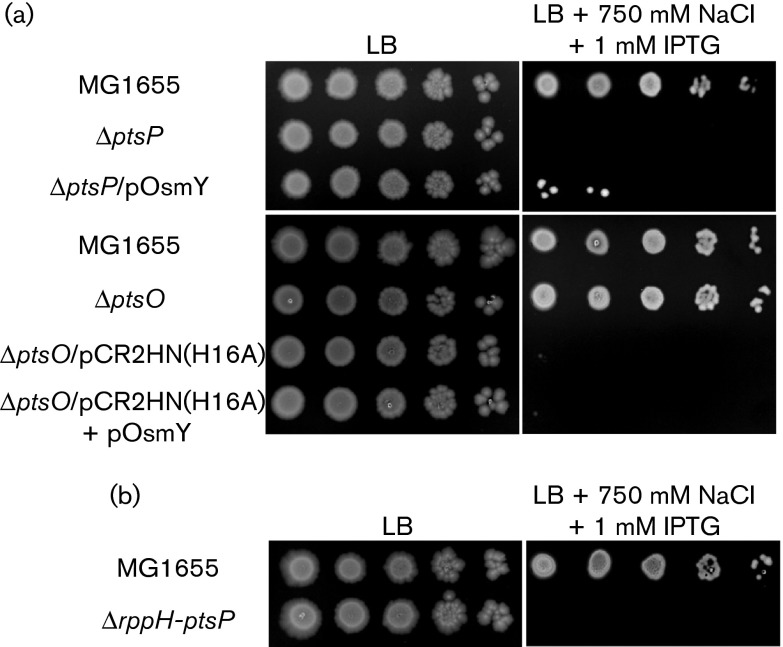
Salt hypersensitivity of cells with increased levels of dephosphorylated NPr is not suppressed by overexpression of the *osmY* gene. (a, b) Stationary-phase cells of the indicated strains were serially diluted 10-fold from 10^8^ to 10^4^ cells ml^−1^ and spotted onto LB plates with and without the addition of 750 mM NaCl and 1 mM IPTG as indicated.

The *ptsP* gene in *E. coli* forms an operon with the upstream gene *rppH* encoding an RNA pyrophosphohydrolase that catalyses conversion of 5′-terminal triphosphate of mRNAs to monophosphate (Deana *et al.*, 2008). However, any physiological connection between the two genes has not been reported until now. A recent study (Lee *et al.*, 2014) showed that the overproduction of RppH renders cells extremely sensitive to high salt, like the *ptsP* mutant, and the salt hypersensitivity of the RppH-overproducing strain was suppressed by overexpression of the *osmY* gene, encoding a periplasmic protein whose expression was inducible under hyperosmotic conditions (Yim & Villarejo, 1992). Therefore, we examined whether the salt-sensitive phenotype of the *ptsP* mutant was also related to the *osmY* gene. Unlike the case of cells overproducing RppH, overexpression of the *osmY* gene could not recover the salt hypersensitivity of the *ptsP* mutant strain (Figs 4a and S4) and deletion of the *ptsP* gene did not affect the expression level of the *osmY* gene (Fig. S5). In addition, the salt hypersensitivity of the dephosphorylated NPr-overproducing strain was also not suppressed by overexpression of the *osmY* gene. Therefore, these results suggested that the salt hypersensitivity of the *ptsP* mutant was independent of the expression level of *osmY*. Although the two genes within the *rppH-ptsP* operon are involved in the same phenotypic feature, their molecular mechanisms seem to be different. This conclusion is also supported by the fact that, despite the significantly increased expression level of *osmY* in the *rppH* mutant and the *rppH ptsP* double mutant, compared with WT and the *ptsP* mutant (Fig. S5), the *rppH ptsP* double mutant was as sensitive to salt stress as the *ptsP* mutant (Fig. 4b), whereas the *rppH* mutant was as resistant to salt stress as WT (Lee *et al.*, 2014).

### The dephospho-form of NPr also affects ethanol stress and SDS stress

In addition to salt stress, the effect of other osmotic stress such as sucrose was investigated. The *ptsP* mutant was also significantly sensitive to sucrose stress, whereas the *ptsO* or *ptsN* mutants exhibited a growth rate similar to that of WT (Fig. S6a). Phenotypes of the double mutant strains of the nitrogen PTS at sucrose stress were similar to those at salt stress (Fig. S6b). Additionally, cells expressing NPr(H16A) were extremely sensitive to sucrose stress, like the *ptsP* mutant (Fig. S6c), indicating that the dephospho-form of NPr regulates various osmotic stresses. The sensitivity to this stress was also independent of NPr-mediated LpxD inhibition (Fig. S6d).

**Fig. 5.**
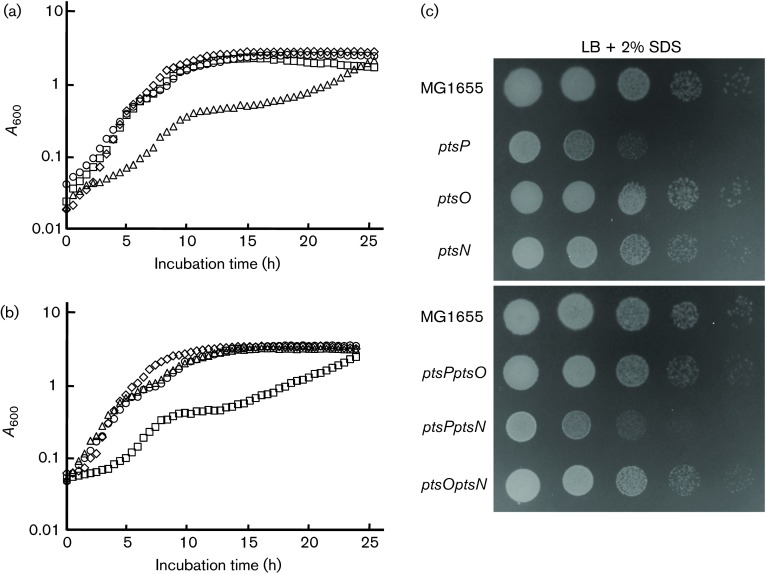
Sensitivity of cells with increased levels of dephosphorylated NPr to ethanol or SDS stress. (a, b) Stationary-phase cells grown in LB medium were inoculated into LB medium containing 5 % ethanol, and growth was recorded by measuring the optical density at 600 nm: (a) Diamonds, MG1655; triangles, CR101(Δ*ptsP*); squares, CR201(Δ*ptsO*) and circles, CR301(Δ*ptsN*). (b) Diamonds, MG1655; triangles, CR121(Δ*ptsP* Δ*ptsO*); squares, CR131(Δ*ptsP* Δ*ptsN*) and circles, CR231(Δ*ptsO* Δ*ptsN*). (c) Stationary-phase cells of the indicated strains were serially diluted 10-fold from 10^8^ to 10^4^ cells ml^−1^ and spotted onto LB plates with the addition of 2 % SDS.

Besides osmotic stress, we analysed the growth rate of the *ptsP* mutant under other stress conditions. We found that the *ptsP* mutant was also sensitive to ethanol and SDS stresses (Fig. 5). Experiments using single mutants and double mutants of the nitrogen PTS genes showed that these phenotypes were also due to the increase of dephosphorylated NPr. Strains with increased levels of dephosphorylated NPr were sensitive to these stresses (Figs S7 and S8). Like the case of osmotic stress, phenotypes sensitive to these stresses were also independent of NPr-mediated LpxD inhibition (Figs S7c and S8). Thus, these results suggested that dephosphorylated NPr negatively regulates the adaptation of cells to envelope stresses, such as osmotic, ethanol and SDS stresses, through an unknown mechanism.

### The C-terminal region of NPr is important to the effect on envelope stresses

**Fig. 6.**
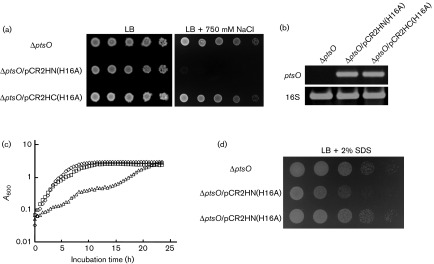
Importance of the C-terminal region of NPr to an envelope stress response. (a) Stationary-phase cells of the indicated strains grown in LB medium were serially diluted 10-fold from 10^8^ to 10^4^ cells ml^−1^, and 2 µl aliquots were spotted onto LB plates with and without the addition of 750 mM NaCl as indicated. (b) The transcript levels of *ptsO* were analysed by RT-PCR with primers specific for *ptsO* or 16S rRNA. The total RNA was extracted from cells grown to mid-exponential phase in the LB media. The 16S rRNA transcript was used as a loading control. (c) Stationary-phase cells grown in LB medium were inoculated into LB medium containing 5 % ethanol, and growth was recorded by measuring the optical density at 600 nm: diamonds, CR201(Δ*ptsO*); triangles, CR201(Δ*ptsO*) transformed with pCR2HN(H16A); squares, CR201(Δ*ptsO*) transformed with pCR2HC(H16A). (d) Stationary-phase cells of the indicated strains were serially diluted 10-fold from 10^8^ to 10^4^ cells ml^−1^ and spotted onto a LB plate with the addition of 2 % SDS.

**Fig. 7.**
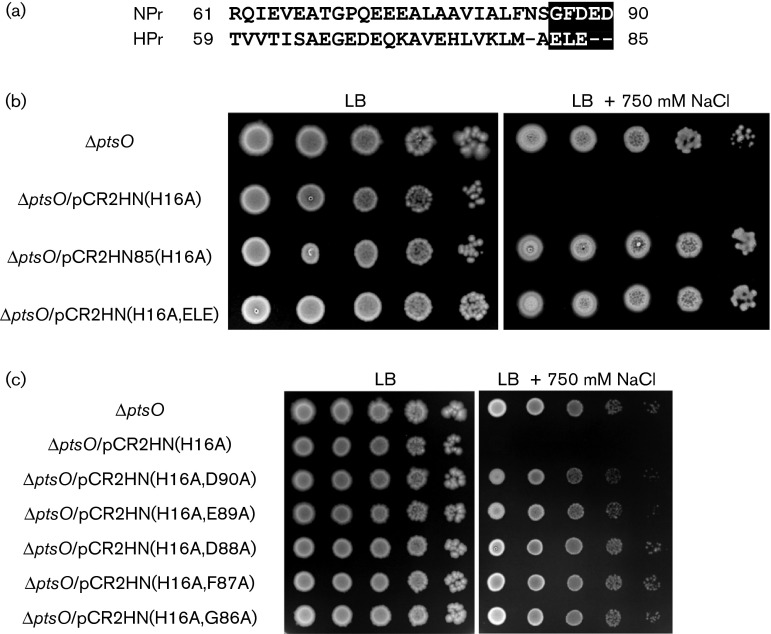
Importance of the C-terminal five residues of NPr to an envelope stress response. (a) Alignment of the C-terminal amino acid sequences of NPr and HPr. Mutated residues are shown in reverse shading. (b, c) Stationary-phase cells of the indicated strains grown in LB medium were serially diluted 10-fold from 10^8^ to 10^4^ cells ml^−1^, and 2 µl aliquots were spotted onto LB plates with and without the addition of 750 mM NaCl as indicated.

During the purification of His-tagged NPrs using both pRE1-based and pET-based expression vectors, we found that an N-terminally His-tagged version of NPr (His-NPr) was significantly insoluble, whereas NPr with six C-terminal histidines (NPr-His) was highly soluble (did not make an inclusion body) and was easier to purify than His-NPr (data not shown). These results implied that the intracellular feature of NPr-His may be considerably different from that of His-NPr. To explore this assumption, we constructed two pRE1-based plasmids, a pCR2HN(H16A) plasmid expressing His-NPr(H16A) and a pCR2HC(H16A) plasmid expressing NPr(H16A)-His. Like cells expressing NPr(H16A) without a His tag, the cells expressing His-NPr(H16A) were sensitive to envelope stresses (Fig. 6). However, expression of NPr(H16A)-His did not exhibit any effect on the adaptation to these stresses, despite similar expression levels of NPr in these two strains (Figs 6b and S9a). A sequence alignment of NPr with HPr showed that the C-terminus region of the two proteins was significantly different (Fig. 7a). Because overexpression of wild-type NPr was problematic, the three-dimensional structure of a truncated form constructed by deleting the five C-terminal residues (NPr85) has been determined (Li *et al.*, 2008). These results suggested that these five C-terminal residues could be important in determining the structural feature of NPr. Therefore, we constructed two plasmids, pCR2HN85(H16A), expressing His-NPr85(H16A) truncated at residue 85, and pCR2HN(H16A,ELE), expressing His-NPr(H16A,ELE) where the five C-terminal residues (GFDED) of NPr were replaced with the three C-terminal residues (ELE) of HPr. The changes of these five amino acids entirely abolished the negative effect of dephosphorylated NPr on salt stress (Fig. 7b). To further explore the importance of these five residues in the C-terminal region, we performed systematic site-directed mutagenesis in this region by changing each residue, one at a time, to alanine. Notably, none of the strains expressing mutant proteins were sensitive to salt stress (Fig. 7c) even though all of these proteins were expressed at sufficiently high levels (Fig. S9b). These results implied that the C-terminal region of NPr was important for adaptation to envelope stress.

## Discussion

In this study, we investigated a novel cellular role of dephosphorylated NPr on envelope stresses. Phenotype analyses using single and double mutants of the nitrogen PTS genes provided us with a clue that the dephospho-form of NPr is involved in sensitivity to envelope stresses, such as osmotic, ethanol and SDS stress. The *ptsP* mutant became extremely sensitive to salt stress, whereas *ptsO* and *ptsN* mutants exhibited normal growth under high salt conditions (Fig. 1). Growth inhibition of the *ptsP* mutant on salt stress was recovered by additional deletion of the *ptsO* gene, but not by additional deletion of the *ptsN* gene, suggesting that an increased level of dephosphorylated NPr in the *ptsP* mutant renders cells sensitive to salt stress (Fig. 2b). Further experiments using cells harbouring a pNPr(H16A) plasmid expressing the unphosphorylatable form of NPr confirmed this assumption (Figs 2c and S2). A negative effect of dephosphorylated NPr was also shown in sucrose, ethanol and SDS stresses (Figs 5 and S6).

Together with our results, several lines of evidence support the connection between the envelope stress response and the nitrogen PTS. From an accurate promoter prediction model and the upregulation upon overexpression of *rpoE*, it was suggested that *ptsO* and *ptsN* genes, together with the *rpoN* gene, are under the control of the extracytoplasmic stress sigma factor, σ^E^ (Rhodius *et al.*, 2006). Another report showed that overexpression of the phosphorylated form of EIIA^Ntr^ suppresses the essentiality of σ^E^ (Hayden & Ades, 2008). Notably, the phosphorylated form of EIIA^Ntr^ reduced extracytoplasmic stress, whereas the dephosphorylated form of NPr increased envelope stress. The dephosphorylated form of EIIA^Ntr^ regulates homeostasis of potassium (Lee *et al.*, 2007; Lüttmann *et al.*, 2009; Pflüger-Grau & Görke, 2010), which is an important cellular ion involved in the adaptation to osmotic conditions and the maintenance of cell turgor (Epstein, 2003). It was also shown that the dephosphorylated form of NPr inhibits the biosynthesis of LPS (Kim *et al.*, 2011), which is also important in maintaining the integrity of the bacterial cell envelope (Vuorio & Vaara, 1992). Although the reason why the nitrogen PTS located in the same operon with *rpoN* is involved in the envelope stress response remains obscure, a recent report using a comparative genome analysis suggested that the enigmatic sigma factor σ^54^ is a central controller of the bacterial exterior (Francke *et al.*, 2011). Therefore, the relationship between the *rpoN* operon including the nitrogen PTS and the envelope stress response needs to be investigated in more detail.

From experiments using double deletion mutants of the nitrogen PTS genes, we discovered that the growth defect of the *ptsP* mutant is caused by increased levels of the dephospho-form of NPr. Mutation of the *ptsP* gene in various bacteria affected various physiological processes, including virulence (Higa & Edelstein, 2001), pyocyanin production (Xu *et al.*, 2005), susceptibility to opsonization (Zhang *et al.*, 2005), dimethyl sulfone utilization (Kouzuma *et al.*, 2007) and root colonization (Mavrodi *et al.*, 2006), but its molecular mechanism was not elucidated. Our results propose that some of these phenotypes of the *ptsP* mutant might be caused by the dephospho-form of NPr.

This study also demonstrated that the change in the C-terminal region of NPr abolishes its capability to negatively regulate the adaptation to envelope stresses. Because of the difficulty in overexpression and purification of wild-type NPr, the three-dimensional structure of NPr has been determined only in modified forms at the C terminus, a form fused with an intein sequence to a chitin-binding domain (Wang *et al.*, 2005) and a truncated form made by deleting the C-terminal five residues (NPr85) (Li *et al.*, 2008). These three-dimensional structures of NPr showed that the C-terminal region of NPr is located close to the phosphorylation site (H16). This fact might explain the reason why the C-terminal region of NPr plays an important role in the phosphorylation-dependent response of NPr to envelope stresses. In addition, heteronuclear nuclear Overhauser effects (NOEs) values for NPr85 showed that two residues at the C terminus of NPr85 have increased motions (Li *et al.*, 2008), which have been shown to be essential for protein–protein interactions (Hansen *et al.*, 2008; Peterkofsky *et al.*, 2001). These results imply that a partner protein(s) of NPr may bind to the C-terminal region in a phosphorylation-dependent manner.
